# Biochemical characteristics of patients with imported malaria

**DOI:** 10.3389/fcimb.2022.1008430

**Published:** 2022-11-10

**Authors:** Dewu Bi, Jianyan Lin, Xiaolu Luo, Lü Lin, Xike Tang, Xiaocheng Luo, Yuexi Lu, Xiaodong Huang

**Affiliations:** ^1^ Department of Clinical Laboratory, Fourth People’s Hospital of Nanning, Nanning, Guangxi, China; ^2^ Affiliated Infectious Disease Hospital of Nanning, Guangxi Medical University, Nanning, Guangxi, China

**Keywords:** imported malaria, biochemical outcomes, risk factor, parasite clearance, prognostic model

## Abstract

**Objectives:**

This study aimed to investigate the clinical and biochemical profiles of patients with imported malaria infection between 1 January 2011 and 30 April 2022 and admitted to the Fourth People’s Hospital of Nanning.

**Methods:**

This cohort study enrolled 170 patients with conformed imported malaria infection. The clinical and biochemical profiles of these participants were analyzed with malaria parasite clearance, and signs and symptoms related to malaria disappearance were defined as the primary outcome. A multivariable logistic regression model was used to evaluate the odds ratios (ORs) with 95% confidence intervals (CIs) for cerebral malaria. The Cox model was used to estimate the hazard ratios (HRs) with 95% CIs for parasite clearance.

**Results:**

Adenosine deaminase and parasitemia were found to be independent risk factors for severe malaria in patients with imported malaria (OR = 1.0088, 95% CI: 1.0010–1.0167, *p* = 0.0272 and OR = 2.0700, 95% CI: 1.2584–3.4050, *p* = 0.0042, respectively). A 0.5–standard deviation (SD) increase of variation for urea (HR = 0.6714, 95% CI: 0.4911–0.9180), a 0.5-SD increase of variation for creatinine (HR = 0.4566, 95% CI: 0.2762–0.7548), a 0.25-SD increase of variation for albumin (HR = 0.4947, 95% CI: 0.3197–0.7653), a 0.25-SD increase of variation for hydroxybutyrate dehydrogenase (HR = 0.6129, 95% CI: 0.3995–0.9402), and a 1.0-SD increase of variation for ferritin (HR = 0.5887, 95% CI: 0.3799–0.9125) were associated with a higher risk for increased parasite clearance duration than a low-level change.

**Conclusions:**

Aspartate aminotransferase, urea, creatinine, albumin, hydroxybutyrate dehydrogenase, and ferritin are useful biochemical indicators in routine clinical practice to evaluate prognosis for imported malaria.

## Introduction

Malaria, caused by Plasmodium and transmitted by mosquitoes (Phillips et al., 2017; Walter and John, 2022), is an epidemic infectious disease that predominantly occurs in tropical and subtropical regions (Chaves et al., 2020; van Dorp et al., 2020; Zhou et al., 2020). Globally, there are over 4.72 billion confirmed cases with approximately 11 million deaths over the past two decades, as reported by the World Health Organization (WHO, 2021). China has succeeded in controlling indigenous malaria, and no autochthonous cases have been reported since 2017 (Burki, 2021; Zhou, 2021). However, with the increasing globalization and interconnectedness, an increasing number of people travel to and from malaria-endemic regions. Therefore, the incidence of imported malaria has been gradually increasing and has become a major public health challenge (Feng et al., 2014; Wang et al., 2018; Feng et al., 2020; Li et al., 2021; Liu et al., 2021).

Human malaria, one of the most lethal infectious diseases responsible for high morbidity and mortality (Dos-Santos et al., 2014; Phillips et al., 2017; Ashley et al., 2018; Chaves et al., 2020), is characterized by circulatory inflammatory events and impairment of the microvascular endothelium (Burté et al., 2013; Morrell, 2014; O’Sullivan and O’Donnell, 2018; Erice and Kain, 2019; Knackstedt et al., 2019; Yeo et al., 2019; Mita Mendoza et al., 2020; Neida, 2020; Vasquez et al., 2021). The pathogenesis of malaria is multifactorial and driven predominantly by parasite biomass and modulation by host innate and adaptive immune responses (Yeo et al., 2008; Barber et al., 2015; Barber et al., 2017; Kho et al., 2018; Cao and Vickers, 2021; Dassah et al., 2022). During infection, red blood cells are invaded by merozoites, which grow and develop by schizogony, generating more merozoites during the intraerythrocytic developmental cycle. These merozoites undergo multiple successive rounds of cell division and are released into the blood stream with their metabolites. This triggers the host complex immune response and disrupts the intracellular environment. Immune cell activation triggers host defense, which limits pathogen spread at the site of infection (Vaid, 2010; Miller et al., 2013; Morrell et al., 2014; Niang et al., 2014; Phillips et al., 2017; Mather and Ke, 2018). Host defense to malaria includes a cascade of pathways that are modulated by hundreds of immune modulatory molecules (Miller et al., 2013; Niang et al., 2014; Gramaglia et al., 2017; Phillips et al., 2017). Many of these molecules promote vasculature occlusion for inducing inflammation and endothelial cell activation and induce acute-phase responses to prevent the spread of malaria. Routine laboratory results indicate liver and kidney dysfunction (Nieman et al., 2009; Nantakomol et al., 2011; Mita-Mendoza et al., 2013; Mangal et al., 2017; Cheaveau et al., 2019; Dinkar et al., 2020; Khrapunov et al., 2021; Tona Lutete et al., 2021; Megabiaw et al., 2022), and in some cases, abnormal myocardial enzyme is reported (Herr et al., 2011; Dinkar et al., 2020; Kaiser et al., 2020).

This study aimed to investigate the biochemical characteristics of 170 patients with imported malaria treated with curative intent in the Fourth People’s Hospital of Nanning using a population-based retrospective cohort.

## Materials and methods

### Study design and participants

In this retrospective study, data were collected and analyzed prospectively from patients with confirmed malaria infection and admitted to the Fourth People’s Hospital of Nanning between 1 January 2011 and 31 May 2022. Only patients with imported malaria were enrolled in this study. Inclusion criteria were as follows: (і) patients with diagnosis of imported malaria, (ii) patients admitted to the hospital within 3 h of onset, and (iii) patients hospitalized with up to three consecutive blood smears being negative with each measurement spaced 24 h, or the disappearance of all clinical evidence of malaria for a minimum of 3 days with no persisting clinical signs and symptoms related to the disease. Imported malaria cases were defined as follows: patients received a diagnosis of malaria, patients had a travel history to a malaria-endemic area, and the onset of symptoms in patients is less than 1 month after returning to China.

Blood samples for microscopic examination and clinical chemistry measurements were collected at the same time, and each collection for microscope examination and clinical chemistry measurement was spaced 24–48 h. Blood samples were centrifuged before routine biochemical tests; biochemical assays were performed using an automatic biochemical analyzer (Roche Modular PPE and Hitachi LABOSPECT 008 AS). Laboratory diagnosis of malaria infection was based on the microscopic examination of Giemsa-stained thin and thick blood smears with confirmed malaria parasites. Additionally, the species of the infecting parasite was confirmed using nested PCR assays.

This study was conducted in accordance with the guidelines of the Declaration of Helsinki and approved by the ethics committee of the Fourth People’s Hospital of Nanning (Nos. [2019]39, [2020]24, and [2021]23).

### Data collection

Patient information, including demographics, epidemiological data, comorbidities, symptoms, laboratory results, and treatment measures, was collected from their electronic medical records. A power analysis was conducted that revealed that a cohort of 170 patients was necessary to achieve more than 80% power based on the primary outcome measure with minimum reference interval.

### Statistical analysis

Normally distributed continuous variables are presented as means and standard deviations (SDs), and non-normally distributed variables are presented as medians and interquartile ranges. Categorical variables are presented as counts (%). The means of continuous variables were compared using independent-samples *t*-tests for normally distributed data, and for other variables, the Mann–Whitney *U*-test was used. Survival analysis was performed using the Kaplan–Meier survival and Cox model analyses. Statistical analyses were conducted using GraphPad Prism software (version 8.0) and MedCalc statistical software (version 15.8). For all statistical tests, *p*< 0.05 was considered to indicate statistical significance.

## Results

### Baseline clinical characteristics

**Figure 1:**
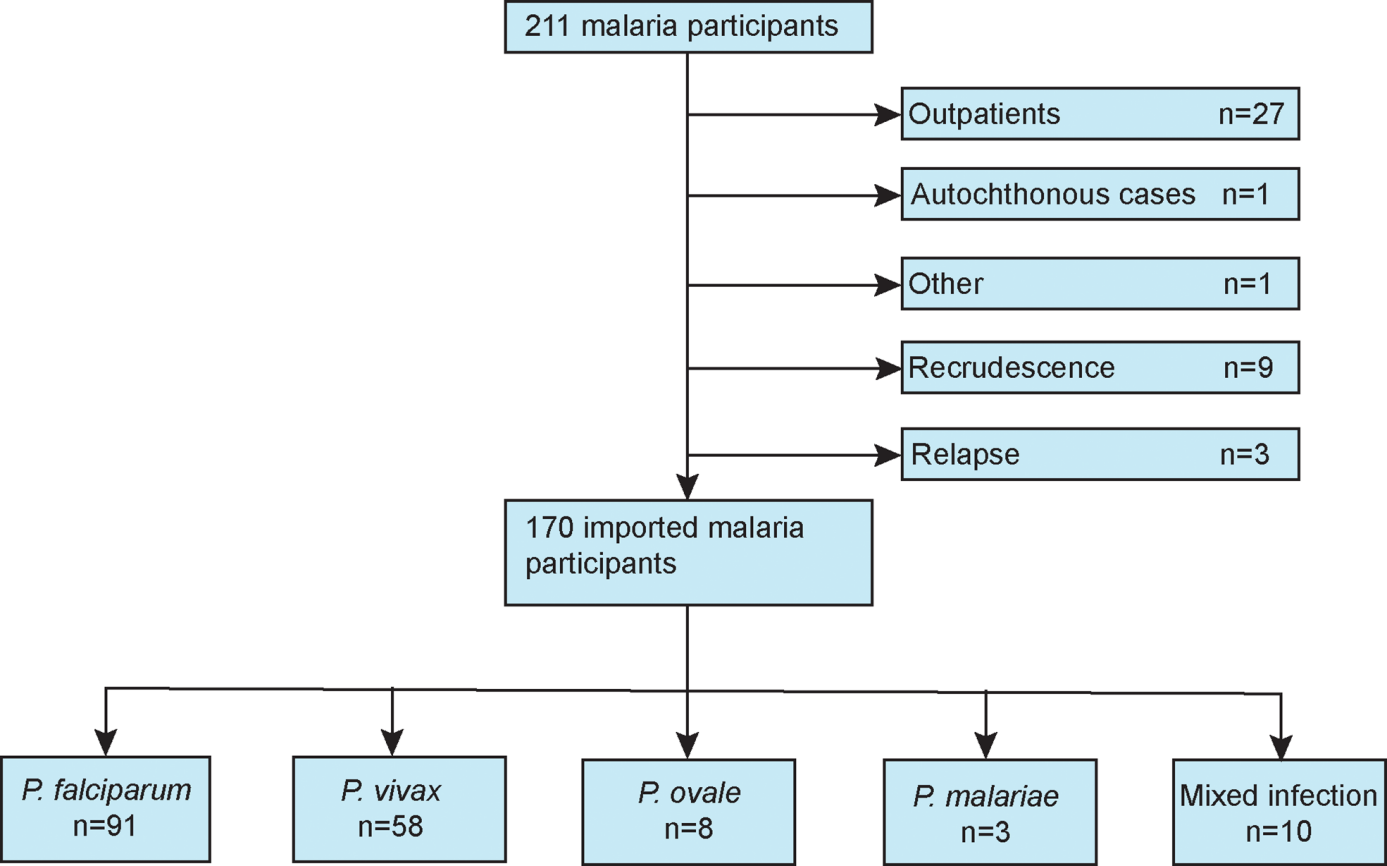
Flow diagram of the patients included in this study.

**Figure 1** presents a flow diagram of the inclusion and exclusion criteria of this study. Demographic, comorbidity, prior malaria history, and treatment variables for patients with imported malaria are summarize in **Table 1**. During the study period, 170 patients with confirmed imported malaria were tested for all indices of serum biochemistry (**Table 2**). Of the participants, 95% were male individuals, and 56 participants (33%) were diagnosed with severe malaria. The criteria for severe malaria are presented in **Table 3**. The results of the analysis of epidemiological data showed that more than 90% cases had recent history of working in an endemic area, primarily in Africa such as Ethiopia, Ghana, and Angola, and more than 70% cases had history of malaria. However, more than 98% cases had history of consuming prophylactic medication with antimalarials.

**Table 1:** Demographics and baseline characteristics of patients with imported malaria.

Parameter	Uncomplicated malaria(n = 114)	Severe malaria(n = 56)	*p*-value
Sex			
Women	4	3	0.6853
Men	110	53	
Age (years)	38 (30–45)	40 (33–46)	0.4421
Occupation			
Worker	105	55	0.1682
Visitor	4	1	0.2177
Traveler	4	0	>0.9999
Student	1	0	>0.9999
Comorbidities			
Hepatitis B virus	2	0	>0.9999
COVID-19	1	0	>0.9999
Diabetes	2	0	>0.9999
History of malaria	77	38	>0.9999
History of treatment	111	47	>0.9999

Age is in median (IQR), the rest are n. p-value was calculated using Fisher’s exact test.

**Table 2:** The biochemical results of patients with imported malaria.

Parameter	Results	Reference limits	Ratio of abnormalities
Urea (mmol/L)	5.3 (4.5–6.5)	3.1–8.0	17%
Creatinine (µmol/L)	86.2 (76.3–98.6)	57.0–111.0	20%
Uric acid (µmol/L)	307.5 (243.3–372.3)	208.0–428.0	23%
Bicarbonate (mmol/L)	24.7 (22.3–26.5)	22.0–32.0	25%
Glucose (mmol/L)	6.1 (5.3–7.8)	3.89–6.11	37%
Creatine kinase isoenzyme-MB (U/L)	15.0 (12.0–20.0)	0.0–25.1	16%
Creatine kinase (U/L)	89.2 (49.7–183.0)	50.0–310.0	36%
Lactate dehydrogenase (U/L)	283.0 (221.5–363.0)	120.0–250.0	61%
Aspartate aminotransferase (U/L)	25.0 (19.0–38.2)	0.0–40.0	22%
Alanine aminotransferase (U/L)	25.0 (18.4–44.0)	0.0–40.0	29%
Total bilirubin (µmol/L)	21.0 (14.4–33.9)	0.0–26.0	38%
Direct bilirubin (µmol/L)	7.4 (4.9–14.1)	0.0–8.0	43%
Globin (g/L)	26.1 (22.4–29.9)	20.0–40.0	2%
Albumin (g/L)	38.9 (33.9–42.8)	35.0–52.0	31%
Ferritin (ng/ml)	800.5 (576.3–1158.0)	20.0–300.0	100%
Adenosine deaminase (U/L)	17.0 (13.0–27.2)	0.0–10.0	87%
Hydroxybutyrate dehydrogenase (U/L)	225.0 (179.0–297.0)	72.0–182.0	74%
Ca^2+^ (mmol/L)	2.1 (2.0–2.2)	2.11–2.52	42%
Blood amylase (U/L)	45.8 (28.5–63.1)	35.0–135.0	29%
Glucose-6-phosphate dehydrogenase (U/L)	1858.0 (1529.0–2198.0)	1300.0–3000.0	20%

Data are median (IQR).

**Table 3:** Predefined criteria for severe malaria.

Criteria	
1: Cerebral malaria	Symptoms included coma, headache, convulsions
2: Shock	Systolic blood pressure of less than 70 mm Hg
3: Acute renal failure	24-h urine volume of less than 400 ml or serum creatinine concentration of less than 265 µmol/L
4: Hemoglobinuria	Positive urine occult blood
5: Pulmonary edema or acute respiratory distress syndrome	Tachypnea, dyspnea, water-bubbling sound
6: Jaundice and liver dysfunction	Direct bilirubin increased significantly
7: Severe anemia	Hematocrit of less than 15% or hemoglobin of less than 50 g/L
8: Disseminated intravascular coagulation	Platelets of less than 100×10^9^/L, fibrinogen of less than 1.5 g/L, fibrin/fibrinogen degradation products of less than 20 mg/L or D-dimer increased, prothrombin time lengthening and shortening of more than 3 s, or activated partial thromboplastin time lengthening of more than 10 s
9: Severe hypoglycemia	Blood glucose of less than 2.2 mmol/L
10: High parasitemia	Erythrocytes infected by parasites of more than 5%, or Giemsa-stained blood smears of *P. falciparum* schizont stage parasites were observed

Patients with malaria suffering from one or more of the following characteristics were defined as severe malaria.

### Laboratory findings

The results of this study suggest that the majority of biochemical outcomes were within the reference range. Moreover, the levels of lactate dehydrogenase, ferritin, adenosine deaminase, and hydroxybutyrate dehydrogenase were observed to be higher than the reference ranges, whereas a slight decrease in the serum calcium ion concentration was observed. Furthermore, ferritin, a unique biochemical parameter, was elevated in all patients with imported malaria (**Table 2**).

### Factors associated with severe malaria in patients with imported malaria

As compared with patients with uncomplicated malaria, the patients with severe malaria had significantly different levels of adenosine deaminase (odds ratio [OR] = 1.0088, 95% CI: 1.0010–1.0167, *p* = 0.0272), creatine kinase isotype MB (OR = 1.0393, 95% CI: 0.9993–1.0809, *p* = 0.0500), direct bilirubin (OR = 1.0278, 95% CI: 1.0028–1.0535, *p* = 0.0293), α-hydroxybutyrate dehydrogenase (OR = 1.0039, 95% CI: 1.0009–1.0069, *p* = 0.0104), lactate dehydrogenase (OR = 1.0035, 95% CI: 1.0012–1.0058, *p* = 0.0029), urea (OR = 1.1121, 95% CI: 1.0002–1.2366, *p* = 0.0495), albumin (OR = 0.9379, 95% CI: 0.8887–0.9897, *p* = 0.01954), and blood calcium (OR = 0.0697, 95% CI: 0.0074–0.6563, *p* = 0.01999). A multivariable logistic regression model identified adenosine deaminase (OR = 1.0115, 95% CI: 1.0030–1.0201, *p* = 0.0080) and parasitemia (OR = 2.0700, 95% CI: 1.2584–3.4050, *p* = 0.0042) as factors associated with severe malaria. The fitting equation is expressed as Equation 1 below:


P=1/{1+Exp[−(−2.8858+0.0114×ADA+0.7275×Parasitemia)]}

(Equation 1) $$\text{p}=0.0007,\text{DdfF}=2,\text{Cox }\&{\text{Snell R}}^{2}=0.1866$$

where ADA is blood adenosine deaminase (U/L), Parasitemia is blood parasitemia (/µl), and df is the degrees of freedom.

### Factors associated with duration of parasite clearance

**Figure 2:**
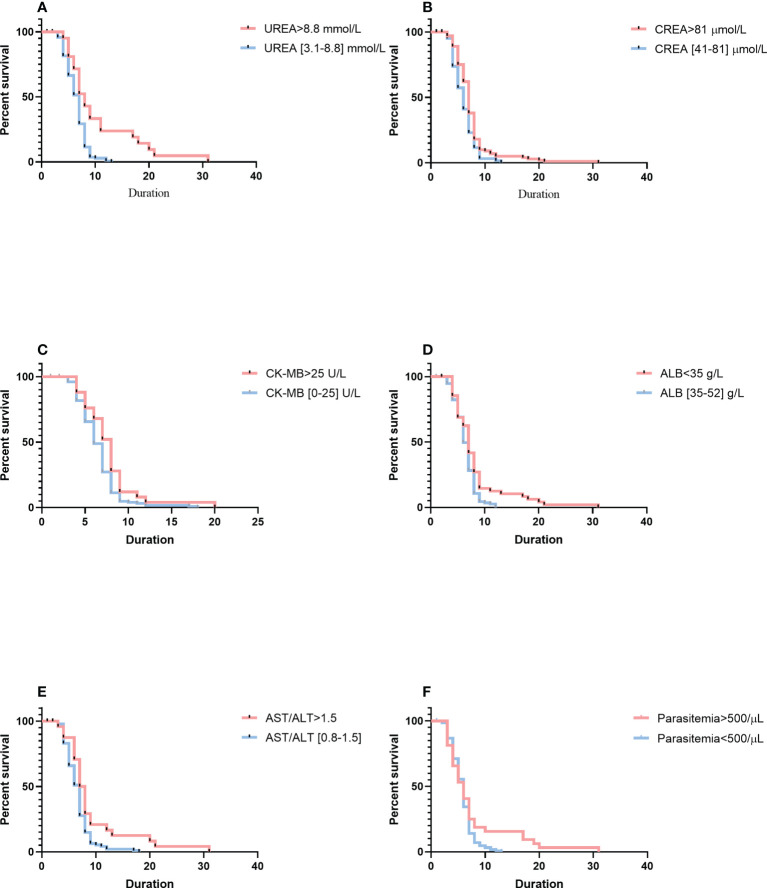
Comparison of survival curves using the log-rank test. **(A)** Serum higher levels of UREA versus within the normal reference range. **(B)** of CREA versus within the normal reference range. **(C)** Serum higher levels of CK-MB versus within the normal reference range. **(D)** Serum lower levels of ALB versus within the normal reference range. **(E)** Serum higher levels of AST/ALT versus within the normal reference range. **(F)** Higher parasitemia versus lower parasitemia. Blue lines show patients with a shorter duration of parasite clearance; red lines show patients with a longer duration of parasite clearance. Duration is indicated in days. CREA, creatinine; CK-MB, creatine kinase isoenzyme-MB; ALB, albumin; AST/ALT, aspartate aminotransferase-to-alanine aminotransferase ratio.

Analysis of parasite clearance risk was performed by using the Cox proportional hazards model (**Figure 2**). Patients with a high level of urea needed longer duration for parasite clearance than those with a normal level of urea (median duration: 8 days, 95% CI: 6–14 *vs*. 6 days, 95% CI: 5–8; *p*< 0.0001). Thus, urea levels were associated with risk for longer parasite clearance duration (hazard ratio [HR] = 0.5108, 95% CI: 0.3533–0.7386). Patients with a high level of creatinine needed a longer duration for parasite clearance (median duration: 7 days, 95% CI: 5–8 *vs*. 6 days, 95% CI: 4–7; *p* = 0.0063) than that of patients who had a normal level of creatinine. Thus, creatinine levels were associated with the risk of a longer length of parasite clearance (HR = 0.6930, 95% CI: 0.4943–0.9718). As compared with patients with normal levels of creatine kinase isoenzyme-MB, patients with higher levels of creatine kinase isoenzyme-MB had a longer duration of parasite clearance (median duration: 8 days, 95% CI: 5–9 *vs*. 6 days, 95% CI: 5–8; *p* = 0.0185). Thus, creatine kinase levels were associated with the risk of a longer length of parasite clearance (HR = 0.6639, 95% CI: 0.4543–0.9702). Patients with a higher ratio of aspartate aminotransferase to alanine aminotransferase had a longer duration of parasite clearance (median duration: 8 days, 95% CI: 6–9 *vs*. 7 days, 95% CI: 5–8; *p* = 0.0050) than that of patients with imported malaria with a normal level of the ratio of aspartate aminotransferase to alanine aminotransferase. Thus, the ratio of aspartate aminotransferase to alanine aminotransferase was associated with the risk of a longer length of parasite clearance (HR = 0.6016, 95% CI: 0.4055–0.8925). However, patients with a low level of albumin had a longer length of parasite clearance (median duration: 7 days, 95% CI: 5–9 *vs*. 6 days, 95% CI: 5–8; *p* = 0.0053) than those with a normal level of albumin. Thus, albumin levels were linked with a higher risk of longer duration of parasite clearance (HR = 0.6850, 95% CI: 0.4985–0.9314). Based on the Cox proportional model, patients with high parasitemia were more likely to have a longer length of parasite clearance (median duration: 7 days, 95% CI: 7–8 *vs*. 4 days, 95% CI: 4–5; *p* = 0.0430) than those with low parasitemia. Thus, parasitemia was associated with a higher risk of longer duration of parasite clearance (HR = 1.364, 95% CI: 0.9557–1.947).

**Figure 3:**
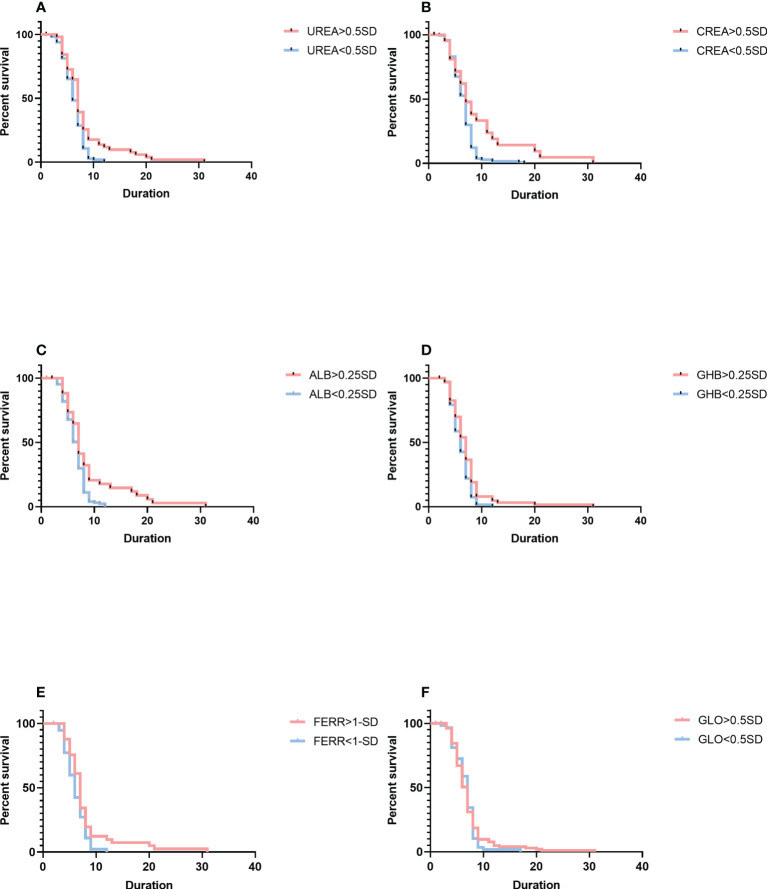
Comparison of survival curves using the log-rank test. **(A)** The variation for UREA ≥ 0.5 SD versus< 0.5 SD. **(B)** The change for CREA ≥ 0.5 SD versus< 0.5 SD. **(C)** The change for ALB ≥ 0.25 SD versus< 0.25 SD. **(D)** The change for GHB ≥ 0.25 SD versus< 0.25 SD. **(E)** The change for FERR ≥ 1.0 SD versus< 1.0 SD. **(F)** The variation for GLO ≥ 0.5 SD versus< 0.5 SD. Blue lines show patients with a shorter duration of parasite clearance; red lines show patients with a longer duration of parasite clearance. Duration is indicated in days. SD, standard deviation; GHB, γ-hydroxybutyrate dehydrogenase; FERR, ferritin; GLO, globin.

More analyses using variation of biochemical indices had been conducted to examine the relationships of blood chemistry with the parasite’s clearance (**Figure 3**). Cox proportional hazard multiple regression analysis suggested that patients with a high level of variation for urea had a longer duration of parasite clearance than those with a low level of variation for urea (median duration: 7 days, 95% CI: 5–9 *vs*. 6 days, 95% CI: 5–8; *p* = 0.0182), and variation for urea was associated with a higher risk of a longer length of parasite clearance (HR = 0.6714, 95% CI: 0.4911–0.9180). Patients with a high level of variation for creatinine had a longer duration of parasite clearance (median duration: 8 days, 95% CI: 5–12 *vs*. 7 days, 95% CI: 5–8; *p* = 0.0444) than patients with a low level of variation for creatinine, and variation for creatinine was associated with a higher risk of a longer length of parasite clearance (HR = 0.4566, 95% CI: 0.2762–0.7548). As compared with patients with a low-level variation of albumin, patients with a high-level variation of albumin had a longer duration of parasite clearance (median duration: 7 days, 95% CI: 5–9 *vs*. 6 days, 95% CI: 5–8; *p* = 0.0285), and albumin levels were associated with a higher risk of a longer length of parasite clearance (HR = 0.4947, 95% CI: 0.3197–0.7653). Patients with a high-level variation of α-hydroxybutyrate dehydrogenase had a longer duration of parasite clearance (median duration: 7 days, 95% CI: 5–8 *vs*. 6 days, 95% CI: 5–7; *p* = 0.0357) than patients with imported malaria with a low level of variation for α-hydroxybutyrate dehydrogenase, and variation for α-hydroxybutyrate dehydrogenase levels was associated with a higher risk of a longer length of parasite clearance (HR = 0.6129, 95% CI: 0.3995–0.9402). Patients with a high level of variation for ferritin had a longer length of parasite clearance (median duration: 7 days, 95% CI: 6–8 *vs*. 6 days, 95% CI: 4–8; *p* = 0.0211) as compared with those who had a low level of variation for ferritin, and variation for the ferritin level was associated with a higher risk of longer duration of parasite clearance (HR = 0.5887, 95% CI: 0.3799–0.9125). A multivariable model based on the variation and the fitting equation was expressed as Equation 2 below:


$$H\left(t\right)=H_{0}(t)\times e^{-0.0225\times \Delta ALB-0.0045\times \Delta CREA-0.0004\times \Delta FERR+0.0002\times \Delta GHB+0.0873\times Par-0.1246\times \Delta UREA}$$

(Equation 2) p=0.0212,chi−square=13.241

where ΔALB is the variation of albumin (g/L), ΔCREA is the variation of creatinine (µmol/L), ΔFERR is the variation of ferritin (ng/ml), ΔGHB is the variation of α-hydroxybutyrate dehydrogenase (U/L), Par is parasitemia (µl), and ΔUREA is the variation of urea (mmol/L).

## Discussion

In this study, biochemical parameters in imported malaria cases were evaluated, and most of these indicators were found to be associated with the severity of the malaria infection. Consistent with previous studies of different biochemical indicators, we found that most parameters in imported malaria are of high abundance, although the opposite is true for a few measurements, and these measures are associated with parasite clearance. Some biochemical parameters were not associated with parasite clearance; however, a high-level variation of these indices is significantly correlated with a longer duration of parasite clearance. Furthermore, a 0.25–1.0 SD increase or decrease of these indicators is associated with a higher risk of a longer length of parasite clearance.

Ferritin, an acute-phase protein in inflammatory responses of various kinds (Damron et al., 2016; Leligdowicz et al., 2021), is the main intracellular iron storage protein in mammals (Li et al., 2012; Truman-Rosentsvit et al., 2018; Fang et al., 2021), plants (Li et al., 2012; Truman-Rosentsvit et al., 2018), and bacteria (Li et al., 2012; Bauckman and Mysorekar, 2016; Truman-Rosentsvit et al., 2018). Moreover, the serum levels of ferritin are strongly associated with infective and non-infective inflammatory conditions (Gabay, 1999; Burté et al., 2013). This study indicated that serum ferritin concentrations did not correlate significantly with parasite clearance, although they were elevated in all patients. Conventionally, serum ferritin has been used as the key iron marker as it reflects the total body iron storage. However, ferritin is also an acute phase protein, so in an acute inflammatory situation, such as during malaria, serum ferritin levels may not accurately reflect body iron stores only. However, a 1-SD increase of variation for serum ferritin levels was associated with a higher risk of duration for parasite clearance, a 1-SD variation of which associates very strongly with malaria parasitemia. Our observations for ferritin are consistent with the findings of previous studies that positively correlated elevated ferritin with the increased risk of malaria parasitemia (Wessells et al., 2017).

In contrast to an increase in the liver enzyme that was observed in previous studies, we observed no elevation of liver enzyme in this study (Viriyavejakul et al., 2014; Rocha et al., 2015; Dinkar et al., 2020; Megabiaw et al., 2022); however, abnormalities in the ratio of aspartate aminotransferase to alanine aminotransferase were observed in 43% of the patients, and higher ratios of aspartate aminotransferase to alanine aminotransferase were significantly associated with malaria parasitemia. The liver enzyme profiles are distinct from prior studies (Viriyavejakul et al., 2014; Dinkar et al., 2020; Megabiaw et al., 2022). Based on the findings on the clinical history of prior prophylactic administration, we speculate that adherence to prophylactic treatment with antimalarial medications before and after infection has a hepatoprotective role. Furthermore, serum albumin values and a 0.25-SD increase of albumin were associated with a significant increased risk of malaria parasitemia. Thus, serum albumin could be a better prognostic risk factor or biomarker for imported malaria than liver enzyme, as abnormal liver function impaired synthesis and acute kidney injury, resulting in an increased albuminuria (Viriyavejakul et al., 2014; Doltario et al., 2016; Leopold et al., 2019; Dinkar et al., 2020).

Multiple studies have shown that malaria is closely associated with hemolysis-induced endothelial activation, resulting in acute kidney injury (Sriboonvorakul et al., 2018; Brown et al., 2020; Kaur et al., 2020; Ouma et al., 2020; Katsoulis et al., 2021). Consistent with previous studies, an increase in the level of creatinine and urea in serum was observed in the case of malaria (Sriboonvorakul et al., 2018; Brown et al., 2020; Kaur et al., 2020; Ouma et al., 2020; Katsoulis et al., 2021). Here, we have described that the plasma levels of renal injury biomarkers correlated with the duration of parasite clearance. Furthermore, in agreement with previous studies of myocardial injury in malaria patients (Dinkar et al., 2020; Watts et al., 2020), we found that most cardiac enzymes in human normal and lactate dehydrogenase increased in malarial infection, with no significant association between lactate dehydrogenase and the duration of parasite clearance. However, the serum levels of creatine kinase isoenzyme-MB, but not creatine kinase isoenzyme, showed a significant association with the duration of parasite clearance. Furthermore, this study provides the first description of a statistically significant association between hydroxybutyrate dehydrogenase and the duration of parasite clearance.

## Data availability statement

The original contributions presented in the study are included in the article/**Supplementary Material**. Further inquiries can be directed to the corresponding author.

## Ethics statement

This study was conducted according to the guidelines of the Declaration of Helsinki and approved by The Ethics Committee of The Fourth People’s Hospital of Nanning (No. [2019]39, [2020]24, [2021]23). Written informed consent for participation was not required for this study in accordance with the national legislation and the institutional requirements.

## Author contributions

Conceptualization, XH. Clinical validation, JL, LL and XT. Investigation, YL and XL. Data curation and original draft preparation, DB. Review, XL. All authors contributed to the article and approved the submitted version.

## Funding

This study was supported by the Key Research and Development Program of Nanning Municipal Science and Technology Department (grant number 20193008), the Guangxi Zhuang Region Health Department (grant number Z20200979), and the Fourth People’s Hospital of Nanning Youth Foundation (grant number NNSY2021010).

## Acknowledgments

The authors would like to thank the staff of the Nanning Center for Disease Control and Prevention and the Guangxi Center for Disease Control and Prevention.

## Conflict of interest

The authors declare that the research was conducted in the absence of any commercial or financial relationships that could be construed as a potential conflict of interest.

## Publisher’s note

All claims expressed in this article are solely those of the authors and do not necessarily represent those of their affiliated organizations, or those of the publisher, the editors and the reviewers. Any product that may be evaluated in this article, or claim that may be made by its manufacturer, is not guaranteed or endorsed by the publisher.
