# Supplementary material for: Biochemical characteristics of patients with imported malaria
Source: Front Cell Infect Microbiol. 2022 Nov 10;12:1008430. doi: 10.3389/fcimb.2022.1008430 (PMC9686291; doi:10.3389/fcimb.2022.1008430)

Comparison of Biochemical Parameters Between *P. falciparum* And *P. vivax*

Statistically significant differences tested by t tests or Mann–Whitney test with 0.05 significance level for bicarbonate (p=0.0002), lactate dehydrogenase (p=0.0273), aspartate aminotransferase (p=0.0465), total bilirubin (p=0.0423), direct bilirubin (p=0.0419), albumin (p=0.0267), ferritin (p=0.0246), and Ca^{2+} (p=0.0482) between *P. falciparum* and *P. vivax* (Table S1). Patients with *P. falciparum* infected had high levels of lactate dehydrogenase, aspartate aminotransferase, total bilirubin, direct bilirubin, albumin, and ferritin, while Patients with *P. vivax* infected had high levels of bicarbonate and Ca^{2+}. These differences may associate with the mechanisms of a vulnerability to malaria infection that *P. falciparum* invades circulating erythrocytes of any age whereas *P. vivax* prefers to young erythrocytes. *P. falciparum* contributes to a vast hemolysis of erythrocytes, and hemolysis or erythrocytes injury give rise to the release of large quantities of enzymes and hemeproteins. Cox proportional hazards model indicated patients with *P. falciparum* infected had a significantly longer duration of parasite clearance than patients with *P. vivax* infected (median duration of parasite clearance: 7 days [IQR 6-8] and 6 days [IQR 4-7], p=0.0014) and associated with a higher risk of longer duration of parasite clearance (HR=0.6378 [95%CI: 0.4429-0.9185]) (Figure S1).

Comparison of Biochemical Parameters Between Severe And Non-severe Groups

At enrollment urea, creatinine, creatine kinase isoenzyme-MB, lactate dehydrogenase, aspartate aminotransferase, alanine aminotransferase, total bilirubin, direct bilirubin, ferritin, and C-reactive protein were significantly elevated in the severe malaria group compared with the non-severe group. However, as compared to the non-severe group, albumin and Ca^{2+} were significantly lower in the severe malaria group. These results indicated that severe and critically malaria patients may suffer from more liver impairment, renal insufficiency, and cardiac dysfunction when hospitalized for standard antimalarial treatment. Variations of acute phase protein, albumin, ferritin, and C-reactive protein, were more evident in the severe malaria group, thus implying that severe infection can induce a more apparent acute phase inflammation response. Cox proportional hazards model indicated patients with severe infection had a significantly longer duration of parasite clearance than patients with non-severe infection (median duration of parasite clearance: 8 days [IQR 7-11] and 6 days [IQR 5-8], p<0.0001) and associated with a higher risk of longer duration of parasite clearance (HR=0.5205 [95%CI: 0.3697-0.7329]) (Figure S1).

Table S1. Comparison of biochemical indicators between *P. falciparum* and *P. vivax*.

Parameter	*P. falciparum* (n = 91)	*P. vivax* (n = 58)	*p* value
Urea (mmol/L)	5.5 (4.6-6.9)	5.1 (4.5-5.9)	0.1522
Creatinine (μmol/L)	86.1 (73.0-110.6)	86.2 (78.2-91.8)	0.5961
Uric acid (μmol/L)	305.0 (207.7-402.3)	317.9 (217.2-418.6)	0.4464
Bicarbonate (mmol/L)	24.0 (21.5-25.5)	25.4 (24.0-27.4)	0.0002
Glucose (mmol/L)	6.3 (5.5-8.1)	6.2 (5.5-7.6)	0.7096
Creatine kinase isoenzyme-MB (U/L)	16.0 (13.0-22.0)	14.0 (11.5-18.0)	0.0822
Creatine kinase (U/L)	80.0 (47.6-159.0)	101.9 (50.4-208.0)	0.3349
Lactate dehydrogenase (U/L)	297.0 (228.4-442.4)	266.5 (219.6-328.6)	0.0273
Aspartate aminotransferase (U/L)	27.6 (19.6-47.5)	21.0 (18.6-36.2)	0.0465
Alanine aminotransferase (U/L)	25.4 (19.4-53.1)	22.6 (17.6-35.6)	0.0571
Total bilirubin (μmol/L)	26.0 (15.0-38.1)	21.0 (14.5-26.4)	0.0423
Direct bilirubin (μmol/L)	8.7 (5.5-16.8)	6.9 (4.7-10.9)	0.0419
Globin (g/L)	25.9 (21.8-30.0)	26.3 (23.2-29.4)	0.6139
Albumin (g/L)	37.1 (32.4-41.6)	41.0 (36.2-43.9)	0.0267
Ferritin (ng/mL)	882.4 (685.7-1274.0)	729.4 (531.1-1021.0)	0.0246
Adenosine deaminase (U/L)	21.0 (13.0-38.0)	16.0 (13.7-22.5)	0.0519
Hydroxybutyrate dehydrogenase (U/L)	224.1 (180.0-323.0)	222.5 (183.5-281.3)	0.4224
Ca^{2+} (mmol/L)	2.1 (2.0-2.2)	2.2 (2.0-2.2)	0.0482
Blood amylase (U/L)	45.0 (35.0-55.5)	49.1 (33.0-58.7)	0.8933
Glucose-6-phosphate dehydrogenase (U/L)	1849.0 (1506.0-2228.0)	1900.0 (1610.0-2147.0)	0.9573

Data are presented as mean with standard deviation (SD), if normally distributed, or median with interquartile range (IQR).

Table S2. Comparison of biochemical indicators between severe and non-severe.

Parameter	Severe (n = 56)	Non-severe (n = 114)	*p* value
Urea (mmol/L)	7.9 (6.0-10.9)	5.1 (4.5-5.9)	<0.0001
Creatinine (µmol/L)	98.2 (85.4-134.7)	84.0 (75.5-96.1)	0.0004
Uric acid (µmol/L)	315.4 (188.2-442.6)	308.5 (218.8-398.2)	0.7323
Bicarbonate (mmol/L)	24.2 (21.6-26.1)	24.7 (22.5-26.6)	0.1942
Glucose (mmol/L)	6.6 (5.2-8.0)	6.1 (5.4-7.8)	0.9538
Creatine kinase isoenzyme-MB (U/L)	18.0 (13.9-27.0)	15.0 (12.0-19.0)	0.0362
Creatine kinase (U/L)	89.7 (46.9-213.3)	85.6 (49.6-170.7)	0.6192
Lactate dehydrogenase (U/L)	407.1 (251.0-607.1)	273.1 (219.6-352.5)	0.0017
Aspartate aminotransferase (U/L)	38.9 (25.3-89.4)	23.6 (18.7-35.9)	<0.0001
Alanine aminotransferase (U/L)	41.6 (19.9-86.4)	24.1 (18.4-38.0)	0.0067
Total bilirubin (µmol/L)	34.2 (19.4-76.5)	20.3 (13.4-30.2)	0.0010
Direct bilirubin (µmol/L)	18.0 (6.4-47.0)	7.1 (4.8-11.2)	<0.0001
Globin (g/L)	26.0 (21.5-30.5)	26.3 (22.7-29.9)	0.7105
Albumin (g/L)	34.5 (31.5-40.5)	39.4 (34.3-43.8)	0.0045
Ferritin (ng/mL)	1051.0 (787.6-1321.0)	794.5 (543.2-1114.0)	0.0150
Adenosine deaminase (U/L)	28.0 (17.0-49.0)	17.0 (13.0-25.0)	0.0628
Hydroxybutyrate dehydrogenase (U/L)	281.0 (209.0-538.0)	224.0 (178.8-286.5)	0.0255
Ca^{2+} (mmol/L)	2.085 (1.903-2.185)	2.140 (2.040-2.218)	0.0408
C-reactive protein (mg/L)	117.2 (68.9-185.4)	64.2 (30.7-111.9)	0.0015
Glucose-6-phosphate dehydrogenase (U/L)	1648.0 (1153.0-2088.0)	1904.0 (1551.0-2207.0)	0.9573

Data are presented as mean with standard deviation (SD), if normally distributed, or median with interquartile range (IQR).

Figure S1: Comparison of survival curves using log-rank test. (A) Severe malaria group vs. non-severe group. (B) P. falciparum group vs. P. vivax group.

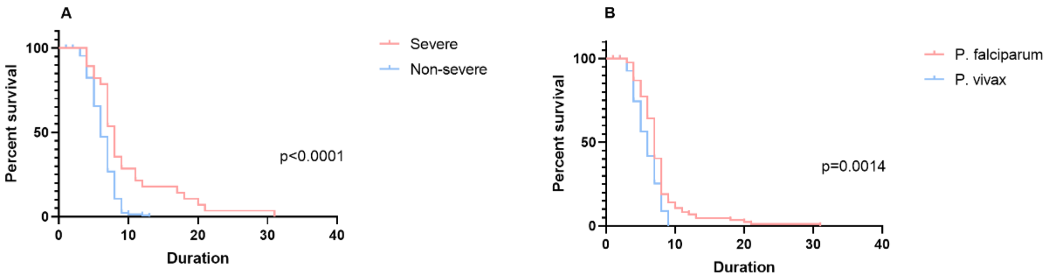

Supplement: Supplementary file 1 [file DataSheet_1.pdf]
